# Duodenal hemangiolymphangioma presenting as chronic anemia: a case report

**DOI:** 10.1186/s13104-016-2214-0

**Published:** 2016-08-31

**Authors:** Sebastian Gómez-Galán, Manuel Santiago Mosquera-Paz, Jorge Ceballos, Paola Andrea Cifuentes-Grillo, Laura Gutiérrez-Soriano

**Affiliations:** 1 General Surgery Program. U. Sabana, Bogotá, Colombia; 2 Fundación Cardioinfantil – Instituto de Cardiología, Bogotá, Colombia; 3 U. Sabana, Bogotá, Colombia

**Keywords:** Hemangioma, Lymphangioma, Small intestine

## Abstract

**Background:**

Lymphangiomas are a heterogeneous group of congenital vascular malformations characterized by cystic dilation of lymphatic vessels. They can occur at any age, but they are more common during childhood and in cutaneous localizations. Hemangiomas and vascular malformations of the gastrointestinal tract are very uncommon. Most are asymptomatic and diagnosis is often made as an incidental finding during endoscopy. On rare occasions the initial manifestation can be chronic anemia due to low grade gastrointestinal bleeding. They constitute an unusual manifestation and there is a low incidence of this type of tumor.

**Case presentation:**

We report the case of a 43 year-old latin female, with a 2-year history of chronic anemia requiring blood transfusion. Hemoglobin and Hematocrit count were low, therefore further studies were required to rule out bleeding sources or other causes of anemia. Enteroscopy findings showed a 35 mm lesion taking up 50 % of the circumference in the distal duodenum, with raised whitish edges secondary to confluent lymphangiectasia, a center with a vascular appearance and active bleeding spots. Biopsy samples dyed with India ink confirmed the diagnosis of hemangiolymphangioma.

**Conclusion:**

Diagnostic difficulties in this case, highlight the need to include hemangiolymphangioma in the differential diagnosis of chronic anemia as well as the need for multiple diagnostic methods to confirm the presence of the condition.

## Background

Hemangiomas and vascular malformations (VM) are uncommon events, even more if they are located in the gastrointestinal tract (GI). The small intestine is the most common location and this type of lesion accounts for 10 % of the benign tumors in the duodenum, jejunum, and ileum. Hemangiomas and VMs of the colon and rectum are the most infrequent with only 200 cases reported from 1931 to 1974 [1]. Diagnosis can be made at any age and 90 % of the cases are diagnosed before age 2. Since March 2011, only one case of pancreatic hemangiolymphangioma (HL) invading the duodenum has been reported, but no reports of HL with small intestine bleeding have been reported [2]. The male/female ratio is 1:2.5; however, when it comes to colon/rectum location, the ratio is 1:1. Even though there are several treatment options, the most effective is surgical resection [3].

## Case presentation

A 43-year-old latin female patient was seen at the gastrointestinal surgery service of Fundación Cardioinfantil—Instituto de Cardiología in Bogotá, with a 2-year history of chronic anemia requiring transfusion of 7 units of packed red blood cells throughout that time span. During the diagnosis work-up for anemia, the patient reported significant genital bleeding during her menstrual period, despite having undergone hysterectomy. Hemoglobin (Hb) and Hematocrit (Htc) count remained low, and thus studies were conducted to explore possible bleeding sources as well as other causes of anemia, such as occult gastrointestinal bleeding.

Laboratory test results at admission to our institution were consistent with microcytic hypochromic anemia (Hb 6,2 gr/dL, Htc 16 %), with negative direct Coombs test and a peripheral blood smear showing no schistocytes. Conditions such as autoimmune hemolytic anemia and paroxysmal nocturnal hemoglobinuria were ruled out as well.

Upper GI endoscopy showed chronic corpus and antrum gastritis and a biopsy confirmed the diagnosis; no evidence of bleeding sites or scars of bleeding foci were found.

**Fig. 1:**
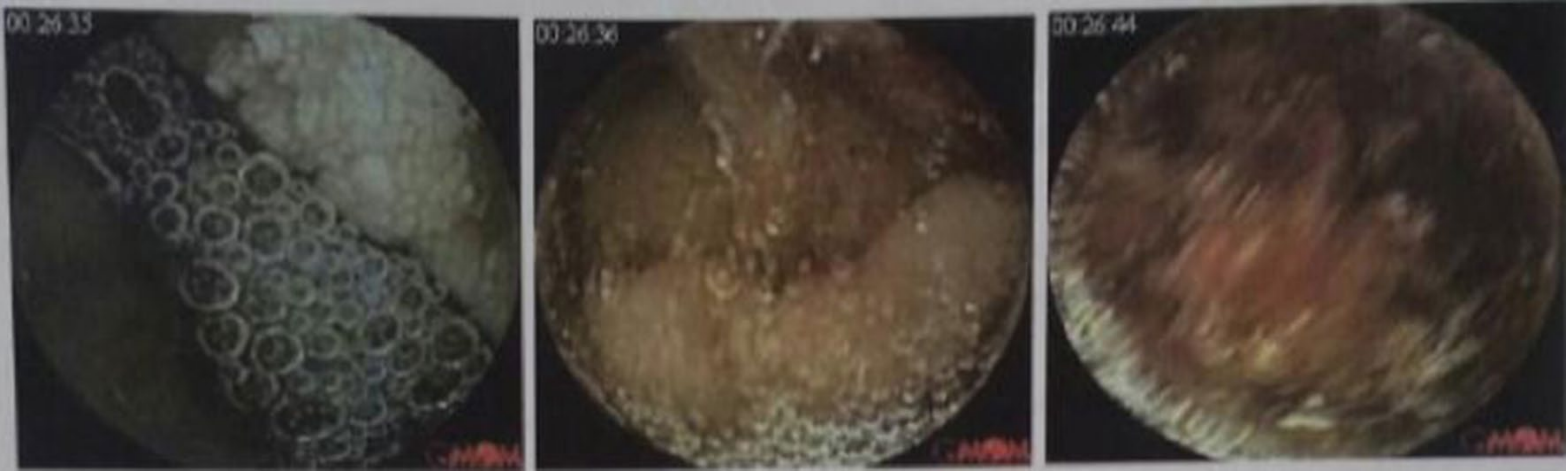
Endoscopic capsule lymphangiectasia and stigmata of bleeding at the distal duodenum and proximal jejunum

Colonoscopy showed scars of previous bleeding in the colon lumen, but no lesions suggesting colonic lesions as the source of anemia. Capsule endoscopy was performed next in order to study hidden causes of intestinal bleeding. Findings showed lymphangiectasia with bleeding stigmas in duodenum 4th portion and jejunum first portion (Fig. 1).

**Fig. 2:**
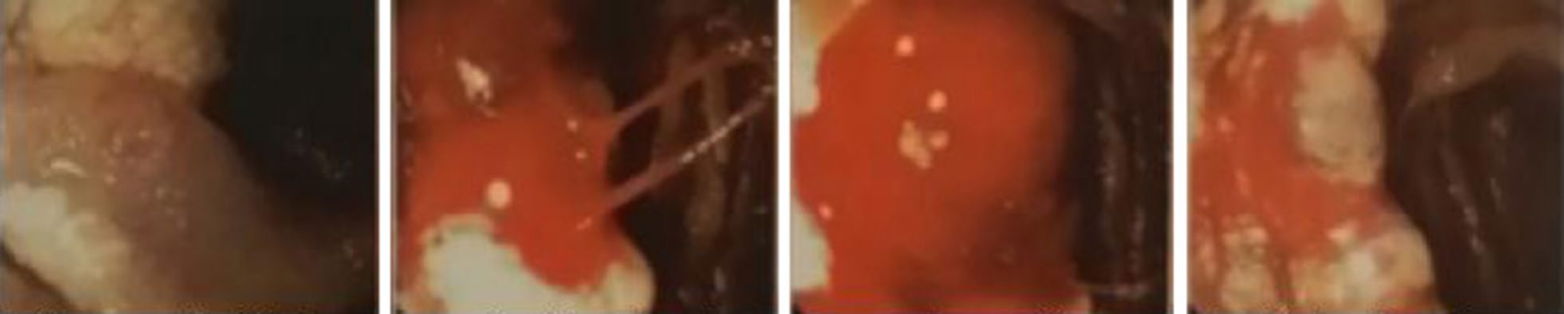
Enteroscopy. Point of active bleeding at the distal duodenum. Biopsy is consistent with hemangio lymphangioma

After this procedure, enteroscopy was carried out, reporting a 35 mm lesion taking up 50 % of the circumference in the distal duodenum, with raised whitish edges secondary to confluent lymphangiectasia, a center with a vascular appearance, and active bleeding spots. Biopsy samples dyed with India ink confirmed the diagnosis of hemangiolymphangioma (Fig. 2).

**Fig. 3:**
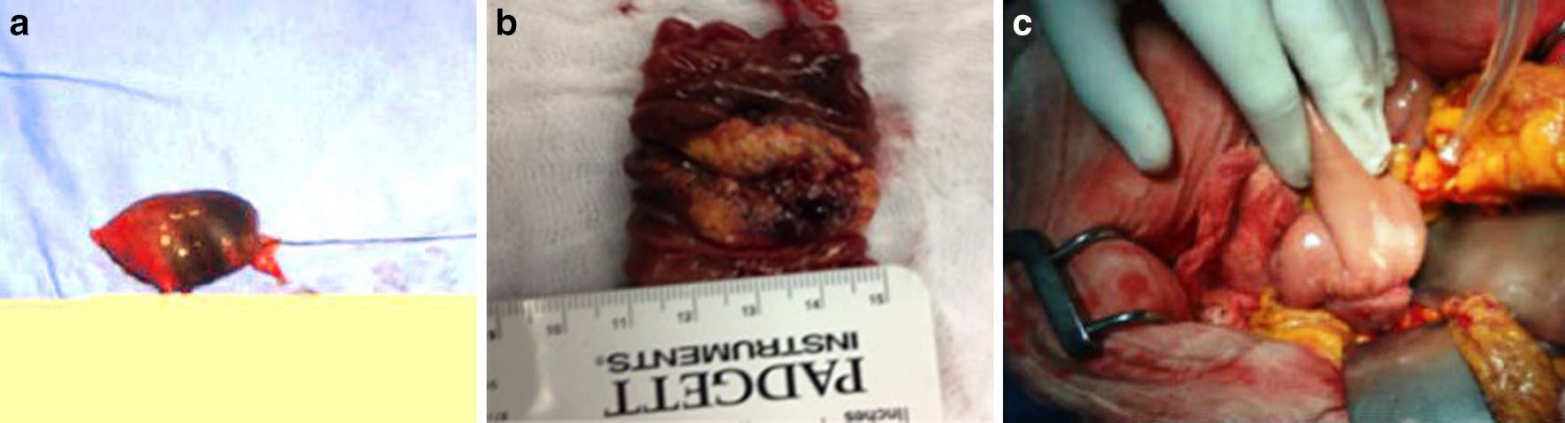
**a** Fourth duodenal segment tattooed with black ink.** b** Surgical specimen removed, showing a 4 × 2 × 1 cm mass with bleeding stigmata.** c** Duodenojejunal lateroterminal anastomosis completed

**Fig. 4:**
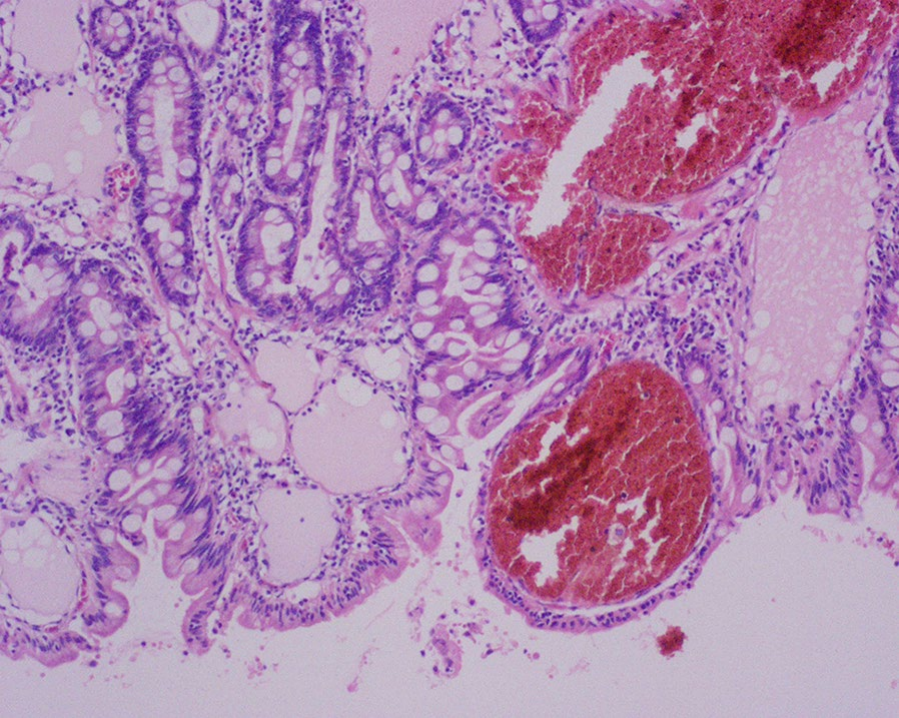
Hemangio lymphangioma with extension to mucosa and submucosa, free edges of injury. H&E 2c ×10

The patient underwent surgical resection of the distal duodenum lesion (fourth portion) plus anastomosis to the first jejunal limb with a side to end technique. Surgery was well tolerated by patient, who received liquid diet 36 h in the postoperative period, patient had a regular evolution and no complications until discharge (Fig. 3). The surgical piece showed a whitish, spongy 4 × 2 × 1 cm lesion, with past bleeding scars (Fig. 4).

## Discussion

In this report, we have described an unusual manifestation of a small intestine hemangiolymphangioma (HL) with chronic anemia due to low grade bleeding. HL is considered a benign disease with no invasive behavior. It is a rare tumor mainly located on the skin. However, cases in the small intestine have been reported as well as in the spleen, thoracic wall, mediastinum, adrenal glands, and colon and rectum [3]. Other terms used for this type of lesions are: arteriovenous malformations, venous or vascular ectasia, and angiodysplasia [4].

These vascular tumors are endothelial neoplasias, characterized by an increase in cellular proliferation. Hemangioma is the most common and almost exclusive of children; it is a fast growing endothelial tumor, with a slow involution with no recurrence. On the other hand, vascular malformations are the result of the abnormal development of vascular structures during embryogenesis. Lymphatic malformations come in various forms, from a localized spongy lesion to a lesion compromising an anatomical region or multiple organs. Radiologically and histologically speaking they are characterized by micro or macrocysts, or both [5]. The incidence of HL varies between 1.2 and 2.8 per every 1000 live births, and the male to female ratio is 1:2.5 [2].

HLs are masses with cystic walls within dilated lymphatic spaces and attenuated by endothelial cells. Inside, they are filled with eosinophilic protein liquids, which may be: chylous, serous, or hemorrhagic, or contained within a collagen stroma. These lymphatic lesions grow from a primordial sac which fails to connect with the rest of the lymphatic system during the embryonic development, and which tends to grow due to muscle contractions and increase intramural pressure. Intramural lymphatic obstruction, endothelial permeability damage, swelling, and congenital absence on lymph have been suggested as other possible causes for the development of intestinal lymphangiomas [6].

In 1982, Mulliken and Glowacki classified vascular lesions within a significant scheme, based on their histological and endothelial cell characteristics. They are entities with high endothelial cell volumes, called hemangiomas. These lesions usually appear at birth and most of them have a spontaneous involution. Steroid or interferon administration accelerates the involution process. In normal endothelial conditions, the correct term is vascular malformations, which are classified according to their dominating abnormality as: arteriovenous malformations, venous malformations, lymphatic malformations, or capillary malformations. In 1996, the International Society for the study of vascular anomalies approved the following classification system [1–7] (Table 1).

**Table 1:** Hemangiomas versus vascular malformations

	Hemangiomas	Vascular malformations
Histology	High volume of endothelial cells	Normal endothelium
Presence at birth	Rarely present	Present
Clinics	Appearing within 6–8 weeks after birth Growth phase: 1–2 years Spontaneous involution	Growth depends on the individual
Diagnosis	Clinical history	Images (Magnetic Resonance Imaging, Computerized Axial Tomography, Ultrasound, angiography)
Treatment	Observation if involution is incomplete Large lesions: surgery, steroids, or gamma-interferon	Depending on location, size, and symptoms. Laser, sclerotherapy, surgery

Table 2 is based on the histological abnormalities of gastrointestinal vascular malformations [1–8] (Table 2).

**Table 2:** Classification of intestinal vascular malformations

Classification of intestinal vascular malformations
Capillary
Cavernous
Localized (polypoid or non-polypoid)
Diffuse infiltrating (expansive)
Mixed
Hemangiomatosis

The capillary subtype is most commonly located on the skin of the perineum, small intestine, and appendix. It is usually single, with no capsule and well defined; half of this subtype is accompanied by ulcers of the mucosa, edema, and swelling. Eighty percent of sigmoid-rectum malformations are of the cavernous type; unlike the first ones, these have large amounts of endothelial cells and multiple layers of them [1–9].

Most are asymptomatic, or show painless hemorrhage of the digestive tract, whether acute, chronic, or recurrent, and may be mistaken for hemorrhoidal pathologies of colon inflammatory disease. Eighty percent of the patients show intraluminal gastrointestinal bleeding symptoms, and 50 % have chronic anemia. Intestinal obstruction is possible but rare [1].

Differential diagnosis includes adenomatous polyps, tumors, hemorrhoids, and other cystic masses such as: intestinal duplications, mesothelial cysts, and pseudocysts. Most of the intestinal HLs are incidental findings, during an endoscopy or a radiological study [3–6].

Diagnosis of small intestine disorders has been difficult through history, but with the appearance of the endoscopic capsule and double-balloon enteroscopy, small intestine lesion management has been revolutionized. Even though the endoscopic capsule allows a full visualization of the digestive tract, its lack of ability to collect biopsies is a disadvantage, as opposed to the three enteroscopy techniques (double-balloon, single balloon, and spiral), which are the techniques of choice for diagnosis and sampling of small intestine lesions [10].

Endoscopically, it is possible to visualize mucosal edema, nodules and vascular congestion, which may be mistaken for inflammatory bowel disease. Biopsy is not recommended due to the high risk of bleeding, but in some cases, it may be necessary [1].

Endoscopic ultrasonography (EUS) is recommended due to the ease of identifying anechoic masses, as well as establishing the depth of the lesions. The use of EUS is limited to lesions located from the esophagus to the duodenum. Magnetic resonance imaging (MRI) and computerized axial tomography (CAT) are useful for defining the extension and compromise caused by the mass, as well as for surgical resection planning [3–11].

Treatment may be medical, endoscopic, or surgical. Medical treatment, carried out during the acute bleeding episode along with hydric resuscitation preserves the patient’s hemodynamic status. For histologically true haemangiomas, corticosteroids are indicated to speed up tumor regression. However, this option is not effective in vascular malformations [1].

Endoscopic approach is ideal for small polypoid lesions that may be addressed through polypectomy and cauterization. Argon as a clotting inducer has been reported as effective against severe hematochezia. Injection of *n*-butyl-2-cyanoacrylate has also been studied, but this option is only indicated when surgical or endoscopic resection is not possible [1].

The treatment of choice, and also the most effective, is surgical resection, especially when the lesion increases in size or applies pressure on the adjacent organs. Surgeons usually remove all tumor material from adjacent organs. Bearing in mind a small or no potential for invasion, local recurrences vary between 10 and 27 % for full resections and 50–100 % for partial resections in the next 2 years [12]. Partial resections are associated with complications such as infections, fistulae and hemorrhage [2–9].

Other treatment options include sclerotherapy, electrocauterization, cryosurgery, and laser; non-surgical techniques can only improve clinical conditions. Angiography and embolization may be used in acute bleeding cases, but there is a risk of new bleeding [3].

## Conclusions

Hemangiolymphangiomas are rare tumors, especially when located in the gastrointestinal tract. Very few reports with the characteristics described in this report have been published in the medical literature. With this report, we want to highlight the need to include these tumors in the differential diagnosis of chronic anemia, as well as the need for multiple diagnostic methods to confirm the presence of the condition.

## Abbreviations

VM: vascular malformations
GI: gastrointestinal tract
HL: hemangiolymphangioma
CAT: computerized axial tomography
MRI: magnetic resonance
US: ultrasonography
